# Energy Drinks and Their Acute Effects on Arterial Stiffness in Healthy Children and Teenagers: A Randomized Trial

**DOI:** 10.3390/jcm11082087

**Published:** 2022-04-07

**Authors:** Pengzhu Li, Guido Mandilaras, André Jakob, Robert Dalla-Pozza, Nikolaus Alexander Haas, Felix Sebastian Oberhoffer

**Affiliations:** Division of Pediatric Cardiology and Intensive Care, University Hospital, LMU Munich, 81377 Munich, Germany; pengzhu.li.extern@med.uni-muenchen.de (P.L.); guido.mandilaras@med.uni-muenchen.de (G.M.); andre.jakob@med.uni-muenchen.de (A.J.); robert.dallapozza@med.uni-muenchen.de (R.D.-P.); nikolaus.haas@med.uni-muenchen.de (N.A.H.)

**Keywords:** energy drinks, arterial stiffness, pediatrics, prevention

## Abstract

Adolescents are the main consumer group of energy drinks (ED). Studies suggest that acute ED consumption is associated with increased peripheral blood pressure. Little is known of the ED-induced effects on arterial stiffness. Therefore, this study aimed to investigate the acute effects of ED consumption on arterial stiffness in healthy children and teenagers by conducting a prospective, randomized, single-blind, placebo-controlled, crossover clinical trial. Study participants (*n* = 27, mean age = 14.53 years) consumed a body-weight-adjusted amount of an ED or a placebo on two consecutive days. Arterial stiffness was evaluated sonographically by two-dimensional speckle tracking of the common carotid artery (CCA) at baseline and up to four hours after beverage consumption. The ED intake led to a significantly decreased peak circumferential strain of the CCA (11.78 ± 2.70% vs. 12.29 ± 2.68%, *p* = 0.043) compared with the placebo. The results of this study indicate that the acute ED consumption might be associated with increased arterial stiffness in healthy children and teenagers. Minors, particularly those with increased cardiovascular morbidity, should be discouraged from ED consumption.

## 1. Introduction

Energy drinks (EDs) are soft drinks that contain high amounts of sugar, caffeine, and other stimulant compounds such as guarana, taurine, or ginseng [1]. EDs were introduced in the 1960s and have become one of the fastest-growing beverages in the soft drink industry after widespread advertising in the 1990s [2]. EDs are particularly popular among teenagers and young adults. According to a review conducted by Seifert et al., 30% to 50% of adolescents and young adults consume EDs [3]. The main function of EDs is marketed as providing fatigue relief, physical performance enhancement, and concentration improvement [4]. However, heavy ED consumption is associated with a series of cardiovascular side effects, including arterial hypertension and arrhythmia in young adults [5,6,7]. Multiple studies demonstrated that heavy ED consumption is linked with increased blood pressure in young adults [5,8,9]. In addition, a recent publication by our department revealed that acute ED consumption significantly raised systolic and diastolic blood pressure in a pediatric cohort [10].

Arterial stiffness refers to the wall rigidity of the large arterial vessels, including the aorta, the carotid arteries, and the cervical arteries [11]. Healthy large arteries have a strong cushioning function. Arterial stiffening caused by aging and multiple cardiovascular risk factors (e.g., smoking, dyslipidemia, diabetes, excess weight) impairs this cushioning function [12]. The stiffness-induced increase in pulse pressure (PP) was shown to be an independent predictor of cardiovascular risk [13].

The current gold standard for the assessment of arterial stiffness is considered to be carotid-femoral pulse wave velocity (cfPWV) measurement [14]. Other techniques, such as performing an ultrasound of the great arteries, have been recently applied to evaluate arterial stiffness: two-dimensional speckle tracking (2DST) is an advanced, non-invasive imaging technique, which has been widely used to analyze left ventricular function [15]. Recently, 2DST has been applied to assess arterial stiffness by tracking the ultrasonic speckles of the arterial wall during systole and diastole. Through the calculation of the vessel’s deformation (strain), arterial stiffness can be visualized [16,17].

Current studies support the hypothesis that caffeine increases arterial stiffness and thus has an impact on the cardiovascular system [18]. To the best of our knowledge, the acute effects of caffeine-containing ED consumption on arterial stiffness have not been investigated yet.

The aim of this study was to evaluate the acute effects of caffeine-containing ED consumption on arterial stiffness in healthy children and teenagers.

## 2. Materials and Methods

### 2.1. Ethical Statement

This study was conducted according to the guidelines of the Declaration of Helsinki and approved by the Ethics Committee of the Ludwig Maximilians University Munich (Munich, Germany) (protocol code: 20-0993, date of approval: 12 January 2021). We obtained prior written informed consent from all study participants. For minor study participants we additionally obtained prior written informed consent from parents or legal guardians.

### 2.2. Study Population

In total, 27 healthy children and teenagers aged 10–18 years were prospectively enrolled for this study. Study participants were examined for eligibility through personal interviews, clinical examination, conventional echocardiography, 24-h Holter ECG, and 24-h blood pressure monitoring before inclusion. The exclusion criteria were as follows: the presence of chronic diseases such as congenital heart disease, arterial hypertension, presence of severe dysrhythmia, family history of sudden cardiac death, allergies to beverage ingredients, regular use of medication with effects on cardiovascular function, regular use of drugs including smoking and alcohol consumption, and pregnancy.

In study participants <18 years, weight classification was assessed according to the body mass index (BMI, kg/m^2^) percentiles (P.) established by Kromeyer-Hauschild et al. [19]. In study participants ≥18 years, normal weight was defined as BMI < 25 kg/m^2^, overweight as BMI ≥ 25 kg/m^2^ but < 30 kg/m^2^, and obesity as BMI ≥ 30 kg/m^2^.

The caffeine consumption behavior of study participants was assessed in accordance with Shah et al.: rare caffeine consumers if <1 caffeinated beverage per month, occasional caffeine consumers if 1 to 3 caffeinated beverages per month, frequent caffeine consumers if 1 to 6 caffeinated beverages per week, and daily caffeine consumers if ≥1 caffeinated beverage per day [5]. In addition, study participants’ ED consumption behavior was investigated as described above.

### 2.3. Study Design

This study was a prospective, randomized, single-blind (study participants), placebo-controlled, crossover clinical trial conducted by the Division of Pediatric Cardiology and Intensive Care, University Hospital, LMU Munich (Munich, Germany), from April 2021 to October 2021. The study was registered in the German Clinical Trials Register (https://www.drks.de/drks_web/DRKS00027580 (accessed on 27 February 2022)).

Detailed information on the study design was described in a recent publication by our department [20]. In short, eligible study participants were randomized into two groups (Group I: day 1: ED, day 2: placebo; Group II: day 1: placebo, day 2: ED) and received either an ED or a placebo drink on two consecutive days. The administered ED amount was adjusted according to the maximal daily caffeine consumption for healthy children and teenagers (3 mg caffeine per kilogram of body weight per day) as recommended by the European Food Safety Authority [21]. The amount of the administered placebo drink was matched with the ED and did not contain typical ED ingredients such as caffeine, guarana, or taurine. ED and placebo had a similar sugar content and taste. The beverages were administered in an identical and masked drinking bottle at room temperature on both study days.

Participants were required not to consume any sources of caffeine or drugs 48 h before and 24 h after study participation. An overnight fast (apart from water) was requested before every study day. Study participants were asked not to consume any food or liquids during each examination duration. Lastly, after complete data collection, to assess blinding quality, study participants were asked to guess on which study day the ED beverage was administered.

### 2.4. Two-Dimensional Speckle Tracking of the Common Carotid Artery

**Figure 1:**
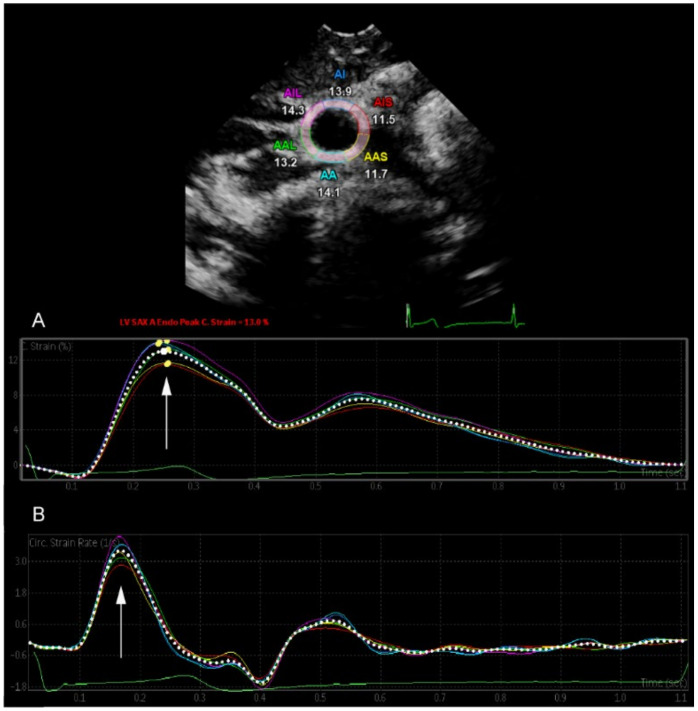
Two-dimensional speckle tracking of the common carotid artery. The arrow indicates (**A**) peak circumferential strain (CS, %) and (**B**) peak strain rate (SR, s^−1^).

An iE33 xMatrix and an Epiq 7G ultrasound machine (Philips, Amsterdam, The Netherlands) were used for examination. Both common carotid arteries (CCA) were recorded in short-axis view just below carotid bifurcation with a 3–8 MHz sector array transducer. During the entire examination period, study participants were in supine position, and the neck was extended to a 45° angle and turned to the opposite side of examination. Three consecutive loops were acquired under constant three-lead ECG tracking. Recorded clips were then transferred to a separate workstation (QLAB cardiovascular ultrasound quantification software, version 11.1, Philips, Amsterdam, The Netherlands). Peak circumferential strain (CS, %) and peak strain rate (SR, s^−1^) of both CCAs were measured semi-automatically through the software’s function “SAX-A”. The vascular region of interest was manually adjusted. Speckles of the vessel wall were then two-dimensionally tracked, as visualized in Figure 1. A masked investigator analyzed the recorded loops three consecutive times, and an average was then calculated. Arterial distensibility (mmHg^−1^ × 10^−3^) was defined as

Arterial distensibility = (2 × Peak Circumferential Strain)**/**(Systolic Blood Pressure − Diastolic Blood Pressure)

Data on ambulatory blood pressure, which were used for the calculation of arterial distensibility, were given in a recent publication by our department [10]. CS, SR, and arterial distensibility of the right and left CCA were averaged.

### 2.5. Endpoints Measurement

The endpoints were CS, SR, and arterial distensibility. For each study day, the endpoints were assessed at baseline as well as 30, 60, 120, and 240 min after beverage consumption.

### 2.6. Statistical Analysis

As this study was a pediatric pilot study, pediatric reference values for ED-induced changes in arterial stiffness did not exist and could not be considered in a power analysis. To test for normal distribution of continuous variables, histograms, QQ-plots, and the Shapiro–Wilk test were applied. Mean and standard deviation were used for all continuous variables. Ordinal and nominal variables are presented as percentages and counts. Sqrt or Ln data transformation was used if data were not normally distributed. A paired *t*-test was applied to compare baseline parameters between the ED and the placebo group. A two-way repeated-measures analysis of variance (ANOVA) was performed to evaluate the main effects of “beverage”, “time”, and interaction of “beverage and time” on CS, SR, and arterial distensibility. The Bonferroni-adjusted pairwise test was used for post hoc testing. Data analyses were performed independently by a masked statistician using SPSS (IBM SPSS Statistics for Windows, version 26.0. IBM Corp., Armonk, NY, USA). A *p*-value < 0.05 was considered statistically significant.

## 3. Results

### 3.1. Patient Characteristics

A total of 27 healthy children and teenagers were included in the analysis. The characteristics of participants are shown in Table 1. None of the subjects had chronic health conditions or was receiving medication. The parameters of arterial stiffness were not significantly different between the two groups at baseline (Table 2). Thirteen of the twenty-seven study participants (48.15%) correctly guessed the day of ED administration, indicating an appropriate blinding quality.

### 3.2. Acute Effects of Energy Drinks on CS, SR, and Arterial Distensibility

The Shapiro–Wilk test revealed a non-normal distribution for the CS at time points baseline, 120 min, and 240 min within the ED group. It revealed a non-normal distribution for the SR at time point 30 min within the placebo group and at time points baseline and 120 min within the ED group. For arterial distensibility, a non-normal distribution was assessed at time points baseline, 60 min, and 120 min within the placebo group and at time points 30 min and 120 min within the ED group. To achieve a normal distribution, the original CS and SR data were transferred into Sqrt-form, and the original arterial distensibility data were transferred into Ln-form. According to Mauchly’s sphericity hypothesis test for the interaction term “beverage and time”, the variance and covariance matrices of the dependent variables were equal (*p* > 0.05).

The interaction between the variables “beverage and time” had no statistically significant effect on the CS, the SR, and arterial distensibility (*p* > 0.05). Hence, the main effect of “beverage consumption” was chosen.

**Figure 2:**
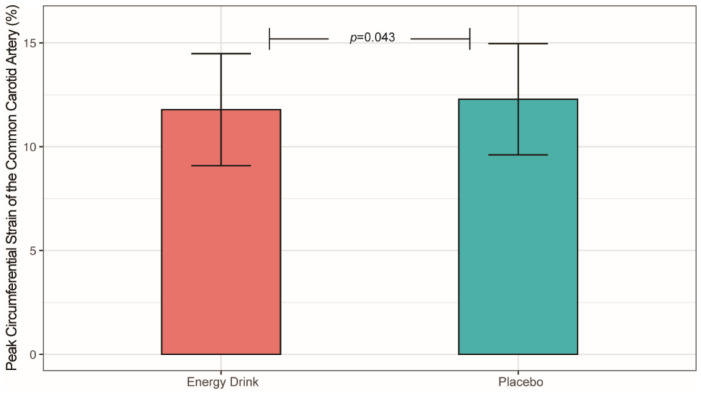
Peak Circumferential Strain (CS, %) of the Common Carotid Artery after Energy Drink and Placebo Consumption.

A two-way repeated-measures ANOVA demonstrated that the CS was significantly lower after ED consumption compared with placebo intake (Table 3, Figure 2). In addition, the SR tended to be lower after ED consumption but did not reach statistical significance (Table 3). Regarding arterial distensibility, no significant differences were assessed between both groups (Table 3).

## 4. Discussion

To the best of our knowledge, this is the first study investigating the acute effects of ED consumption on arterial stiffness in healthy children and teenagers. In total, 27 children with a mean age of 14.53 years were included for strain imaging of the CCA. After ED consumption, a significant decrease in the CS was observed. In addition, the SR tended to be lower after ED intake. Therefore, the results of this study suggest an acute elevation of arterial stiffness in a cohort of healthy children and teenagers after ED consumption.

### 4.1. Pathophysiological Considerations and Clinical Implications

The ED-induced effects on arterial stiffness can mainly be attributed to the high content of caffeine and guarana in EDs. Caffeine is thought to increase peripheral vascular resistance through sympathetic stimulation and consequently effect arterial stiffness [18,22]. Interestingly, a recent publication by our department reported a significant increase in peripheral systolic and diastolic blood pressure in the same pediatric cohort after ED consumption [10]. The results of this study suggest that the increased peripheral vascular resistance may result in an elevation of arterial stiffness visualized by a significant decrease in CS after ED consumption.

For this study, the administered amount of caffeine corresponded to the maximal daily dose (3 mg caffeine per kilogram of body weight per day) recommended for healthy children and teenagers by the European Food Safety Authority [21]. Presumably, the cardiovascular system might respond even more severely to higher amounts of caffeinated EDs. Besides caffeine and guarana, other substances such as taurine, glucuronolactone, and vitamins are commonly added to EDs. It has been suggested that taurine may lower blood pressure and have a positive effect on arterial stiffness [23,24,25,26]. The potential impact of glucuronolactone and vitamins on the cardiovascular system, however, requires further research.

Increased arterial stiffness is associated with elevated cardiovascular risk: the literature suggests that increased arterial stiffness is linked with altered coronary perfusion, elevated left ventricular afterload, left ventricular dysfunction, and left ventricular hypertrophy [12,27]. Therefore, further studies investigating the acute and chronic effects of ED consumption on left ventricular function and morphology are required. Moreover, pediatric studies indicate that increased arterial stiffness leads to structural vascular changes early in life [28]. Besides caffeine, EDs are high in sugar and calories. In particular, chronic ED consumption can increase the risk for glucose metabolism disorders, excess weight, and arterial hypertension. All of these cardiovascular risk factors were shown to be involved in the process of arterial stiffening [11,29,30]. As EDs negatively affect the cardiovascular system, minors, particularly those with already present cardiovascular risk factors (e.g., arterial hypertension, diabetes, excess weight, congenital heart disease), should be discouraged from ED consumption. In the future, studies are needed that evaluate the cardiovascular morbidity, including arterial stiffness, of chronic ED consumers.

### 4.2. Limitations

The limitations of the study design were reported in previous publications of our department [10,20]. The sample size of this study can be considered relatively low, as only 27 study participants were included. As 2DST of the CCA is a relatively new method to evaluate arterial stiffness, pediatric reference values do not exist and could not be elaborated in the current study. In addition, solely healthy children and teenagers were included in the present study. Minors with pre-existing health conditions (e.g., arterial hypertension, congenital heart disease) might react more profoundly to ED ingestion. Further, the relatively small sample size did not allow for an analysis of the influence of sex and habitual caffeine consumption on the parameters studied. Moreover, the ED amount was matched with body weight instead of lean body mass. Lastly, only the acute ED-induced effects on arterial stiffness were investigated, and only one specific ED product was utilized for this study. Hence, further studies are required that take the abovementioned limitations into consideration.

## 5. Conclusions

The acute ED consumption is associated with a significant increase in arterial stiffness in healthy children and teenagers. Minors, particularly those with pre-existing health conditions such as arterial hypertension, diabetes, overweight, or congenital heart disease, should be discouraged from ED consumption. Further studies are required that evaluate the chronic effects of ED consumption on cardiovascular morbidity in children and teenagers.

## Figures and Tables

**Table 1:** Study Participants’ Characteristics (*n* = 27).

Characteristics	Total
Age, years (mean ± SD)	14.53 ± 2.40
Sex, *n* (%)	
Male	14 (51.85)
Female	13 (48.15)
Weight Classification, *n* (%)	
Normal weight	23 (85.19)
Overweight	4 (14.81)
Obese	0 (0)
Caffeine Consumption Behavior, *n* (%) ^a^	
Rare	17 (62.96)
Occasional	3 (11.11)
Frequent	5 (18.52)
Daily	2 (7.41)
Energy Drink Consumption Behavior, *n* (%) ^b^	
Never	12 (44.44)
Rare	11 (40.74)
Occasional	1 (3.70)
Frequent	3 (11.11)
Daily	0 (0)

^a^ Rare caffeine consumer if <1 caffeine-containing drink per month, occasional caffeine consumer if 1 to 3 caffeine-containing drinks per month, frequent caffeine consumer if 1 to 6 caffeine-containing drinks per week, and daily caffeine consumer if ≥1 caffeine-containing drink per day [5]. ^b^ Rare energy drink (ED) consumer if <1 ED per month, occasional ED consumer if 1 to 3 EDs per month, frequent ED consumer if 1 to 6 EDs per week, and daily ED consumer if ≥1 ED per day.

**Table 2:** Parameters of Arterial Stiffness at Baseline (*n* = 27).

Parameters	Energy Drink	Placebo	*p*-Value
CCA CS (%)	12.37 ± 3.01	12.29 ± 2.76	0.89
CCA SR (s^−1^)	3.23 ± 0.73	3.24 ± 0.73	0.94
Arterial Distensibility (mmHg^−1^ × 10^−3^)	538.68 ± 135.25	519.94 ± 117.22	0.49

CCA, common carotid artery; CS, peak circumferential strain; SR, peak strain rate. Mean ± standard deviation were used for normally distributed parameters.

**Table 3:** CS, SR and, Arterial Distensibility after Energy Drink and Placebo Consumption (*n* = 27).

Parameters	Energy Drink	Placebo	*p*-Value
CCA CS (%)	11.78 ± 2.70	12.29 ± 2.68	0.043 *
CCA SR (s^−1^)	3.20 ± 0.73	3.34 ± 0.74	0.087
Arterial Distensibility (mmHg^−1^ × 10^−3^)	504.69 ± 145.50	521.92 ± 134.99	0.313

CCA, common carotid artery; CS, peak circumferential strain; SR, peak strain rate. * *p* < 0.05.

## Data Availability

The data presented in this study are available upon reasonable request from the corresponding author.
